# NP-Scout: Machine Learning Approach for the Quantification and Visualization of the Natural Product-Likeness of Small Molecules

**DOI:** 10.3390/biom9020043

**Published:** 2019-01-24

**Authors:** Ya Chen, Conrad Stork, Steffen Hirte, Johannes Kirchmair

**Affiliations:** 1 Center for Bioinformatics (ZBH), Department of Informatics, Faculty of Mathematics, Informatics and Natural Sciences, Universität Hamburg, 20146 Hamburg, Germany; chen@zbh.uni-hamburg.de (Y.C.); stork@zbh.uni-hamburg.de (C.S.); steffen.hirte@studium.uni-hamburg.de (S.H.); 2 Department of Chemistry, University of Bergen, 5007 Bergen, Norway; 3 Computational Biology Unit (CBU), Department of Informatics, University of Bergen, 5008 Bergen, Norway

**Keywords:** natural products, natural product-likeness, machine learning, random forest, classification, similarity maps, visualization, molecular fingerprints, web service

## Abstract

Natural products (NPs) remain the most prolific resource for the development of small-molecule drugs. Here we report a new machine learning approach that allows the identification of natural products with high accuracy. The method also generates similarity maps, which highlight atoms that contribute significantly to the classification of small molecules as a natural product or synthetic molecule. The method can hence be utilized to (i) identify natural products in large molecular libraries, (ii) quantify the natural product-likeness of small molecules, and (iii) visualize atoms in small molecules that are characteristic of natural products or synthetic molecules. The models are based on random forest classifiers trained on data sets consisting of more than 265,000 to 322,000 natural products and synthetic molecules. Two-dimensional molecular descriptors, MACCS keys and Morgan2 fingerprints were explored. On an independent test set the models reached areas under the receiver operating characteristic curve (AUC) of 0.997 and Matthews correlation coefficients (MCCs) of 0.954 and higher. The method was further tested on data from the Dictionary of Natural Products, ChEMBL and other resources. The best-performing models are accessible as a free web service at http://npscout.zbh.uni-hamburg.de/npscout.

## 1. Introduction

Natural products (NPs) continue to be the most prolific resource for drug leads [1,2,3,4]. A recent analysis found that over 60% of all small-molecule drugs approved between 1981 and 2014 are genuine NPs, NP analogs or their derivatives, or compounds containing an NP pharmacophore [5]. NPs are characterized by enormous structural and physicochemical diversity [6,7,8]. Some of the regions in chemical space covered by NPs are not, or only rarely, populated by synthetic molecules (SMs) [7,9]. The structural complexity of many NPs exceeds that of compounds found in conventional synthetic libraries for screening, in particular with respect to stereochemical aspects, molecular shape, and ring systems [10,11,12,13,14,15,16,17,18].

The primary bottleneck of NP research is the scarcity of materials for testing. In a recent study, we showed that the molecular structures of more than 250,000 NPs have been deposited in public databases, and that only approximately 10% of these are readily obtainable from commercial providers and other sources [19].

Given the fact that NPs exhibit a wide range of biological activities that are of immediate relevance to human health, new avenues that would make NP research more effective are being explored, in particular, research involving computational approaches [2]. For example, computational methods have been employed successfully for the identification of bioactive NPs [20,21,22] and their bio-macromolecular targets [23,24,25,26]. They have also been successfully utilized for the design of simple synthetic, bioactive mimetics of NPs [27,28,29]. In this context, computational methods for quantifying the NP-likeness of compounds can be valuable tools to guide the de novo generation of NP mimetics and optimize the NP-likeness of lead compounds. Such methods may also be useful for identifying genuine NPs in commercial compound libraries, which often also contain SMs [19]. This can be valuable in the context of library design and for the prioritization of compounds for experimental testing.

The best-known in-silico approach for identifying NPs is the NP-likeness score developed by Ertl et al. [30]. The NP-likeness score is a Bayesian measure that quantifies a compound’s similarity with the structural space of NPs based on structural fragments. As such, the model can identify sub-structures characteristic to NPs. The method has been re-implemented, with some modifications, in various platforms (e.g., [31,32,33]). Among them is the Natural-Product-Likeness Scoring System [31], which allows the calculation of the NP-likeness score (with some modifications). The Natural-Product-Likeness Scoring System also allows the use of customized data sets for training. An alternative approach for quantifying NP-Likeness, following a similar modeling strategy, but based on extended connectivity fingerprints (ECFPs), was reported by Yu [34]. Also a rule-based approach has been reported [35].

In this work, we present the development and validation of new machine learning models for the discrimination of NPs and SMs. To the best of our knowledge, these models are trained on the largest collection of known NPs that have been employed for the development of such classifiers. Among further developments, we present the utilization of similarity maps [36] for the visualization of atoms of a molecule, which are characteristic for NPs or SMs, according to the models.

## 2. Materials and Methods

### 2.1. Data Preparation

NPs were compiled from several physical and virtual NP databases (see Results for details). The chemical structures were parsed directly from SMILES notation, where available. Alternatively, chemical structures stored in chemical table files (e.g., SDF) were parsed with RDKit [37] and converted into SMILES. Minor components of salts were removed by the method described in ref. [38]. Any compounds with a molecular weight below 150 Da or above 1500 Da, and any compounds consisting of elements other than H, B, C, N, O, F, Si, P, S, Cl, Se, Br, or I were filtered. The “canonicalize” method, which was implemented in the “tautomer” class of MolVS [39], was used for neutralizing the molecular structures and merging tautomers. After the removal of duplicate SMILES (ignoring stereochemistry), the processed NP reference data set consisted of a total of 201,761 NPs.

SMs were compiled from the “in-stock” subset of ZINC [40,41]. In a first step, 500,000 compounds of ZINC were picked by random selection from the complete “in-stock” subset and pre-processed following the identical protocol used for the NP databases. After generating unique, canonicalized SMILES, any molecules present in the NP reference data set were removed from the SM data set (as determined by the comparison of canonicalized SMILES). Then, random sampling was used to compile a reference data set of SMs of identical size as the NP reference data set (i.e., 201,761 compounds).

The Dictionary of Natural Products (DNP) [42] and the ChEMBL database [43,44] were pre-processed following the identical protocol outlined for the NP and SM data sets. The ChEMBL sub-set of molecules, published in the Journal of Natural Products, was retrieved directly from ChEMBL [43,45]. The natural products subset of ZINC was downloaded from the ZINC website [46].

### 2.2. Principal Component Analysis

Fifteen two-dimensional molecular descriptors calculated with the Molecular Operating Environment (MOE) [47] were used for principle component analysis (PCA): MW (Weight), log *P* (log *P* (o/w)), topological polar surface area (TPSA), number of hydrogen bond acceptors (a_acc), number of hydrogen bond donors (a_don), number of heavy atoms (a_heavy), fraction of rotatable bonds (b_rotR), number of nitrogen atoms (a_nN), number of oxygen atoms (a_nO), number of acidic atoms (a_acid), number of basic atoms (a_base), sum of formal charges (FCharge), number of aromatic atoms (a_aro) and number of chiral centers (chiral), and number of rings (rings).

### 2.3. Model Building

Prior to model building, the preprocessed NP and SM reference data sets were merged, resulting in a total of 403,522 data records. The merged data set was then randomly split into a training set of 322,817 and a test set of 80,705 compounds (ratio of 4:1). In fingerprint space, structurally distinct molecules may have identical fingerprints. For this reason, de-duplication, based on fingerprints, was separately performed for all NPs and all SMs in the training data. Any fingerprints present in both the NP and SM subsets were removed, in order to avoid conflicting class labels. This procedure resulted in a training set of 156,119 NPs and 161,378 SMs represented by Morgan2 fingerprints, and in a training set of 108,393 NPs and 157,162 SMs represented by MACCS keys.

Morgan2 fingerprints (1024 bits) [48,49] and MACCS keys (166 bits) were calculated with RDKit, and 206 two-dimensional physicochemical property descriptors were calculated with MOE. Random forest classifiers (RFCs) were generated with scikit-learn [50,51] using default settings, except for “n_estimators”, which was set to “100”, and “class_weight”, which was set to “balanced”.

The NP-likeness calculator [30,31,52] was trained on atom signatures derived from the identical NP and SM data sets, used for training the RFCs. Subsequently, the NP-likeness score was calculated for each molecule in the test set, according to the atom signatures. All calculations used a signature height of 3, resulting in scores ranging from −3 to 3. Molecules with a score greater than 0.0 were labeled as NPs, and molecules with a score lower, or equal to 0.0 were labeled as SMs. NP class probabilities (and AUCs) were derived by normalizing these scores to a range from 0.0 to 1.0.

### 2.4. Similarity Maps

Similarity maps were computed with the RDKit [37] Chem.Draw.SimilarityMaps module based on RFCs derived from Morgan2 fingerprints (1024 bits).

## 3. Results

### 3.1. Compilation of Data Sets for Model Development

An NP reference data set of 201,761 unique NPs was compiled from 18 virtual NP libraries and nine physical NP databases. The reference data set is identical to that compiled as part of our previous work [8], with two amendments: First, the compounds of the DNP [42] were not included in the data set, as they serve as an external test set in this work, and second, the recently published Natural Products Atlas database [53] was added as a new data source. An overview of the NP data sources utilized in this work is provided in Table 1. The table also reports the number of molecules that are contained in the individual databases prior to, and after, data preprocessing. This is a procedure that includes the removal of salt components and stereochemical information, the filtering of molecules composed of uncommon elements, and with a molecular weight (MW) below 150 Da or above 1500 Da, and the removal of duplicate molecules (see Methods for details). An equal amount (i.e., 201,761) of synthetic organic molecules (SMs) was collected from the “in-stock” subset of ZINC [41] by random selection.

### 3.2. Analysis of the Physicochemical Properties of Natural Products and Synthetic Molecules

**Figure 1:**
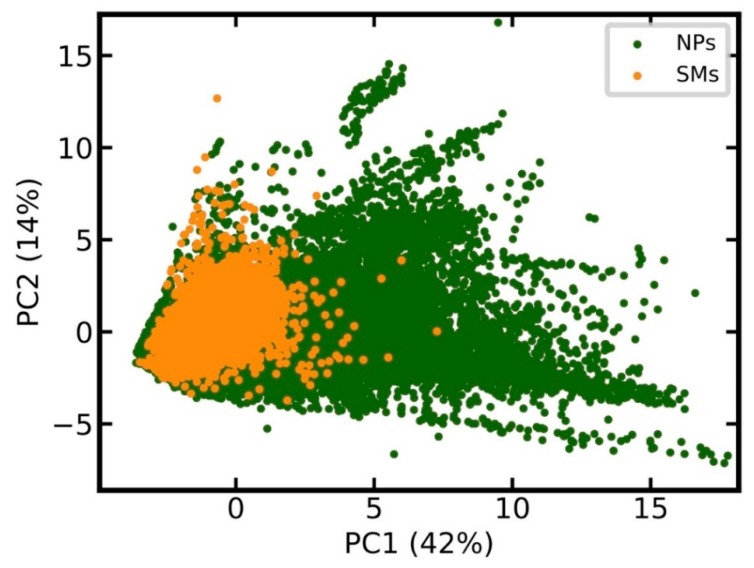
Comparison of the chemical space covered by natural products (NPs) and synthetic organic molecules (SMs). The score plot is based on the principle component analysis (PCA) of all molecules in the data set, characterized by 15 calculated physicochemical properties. PCA was performed on the full data sets. For the sake of clarity, only a randomly selected 10% of all data points are reported in the score plot. The percentage of the total variance explained by the first two principal components is reported in the respective axis labels.

Prior to model development, we compared the chemical space covered by the 201,761 unique NPs, and the equal number of unique SMs, using principal component analysis (PCA), based on 15 relevant physicochemical properties (see Methods for details). The score plot in Figure 1 shows that the chemical space of SMs is essentially a sub-space of NPs.

**Figure 2:**
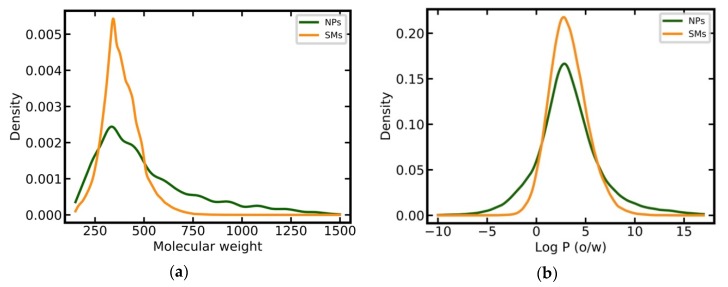

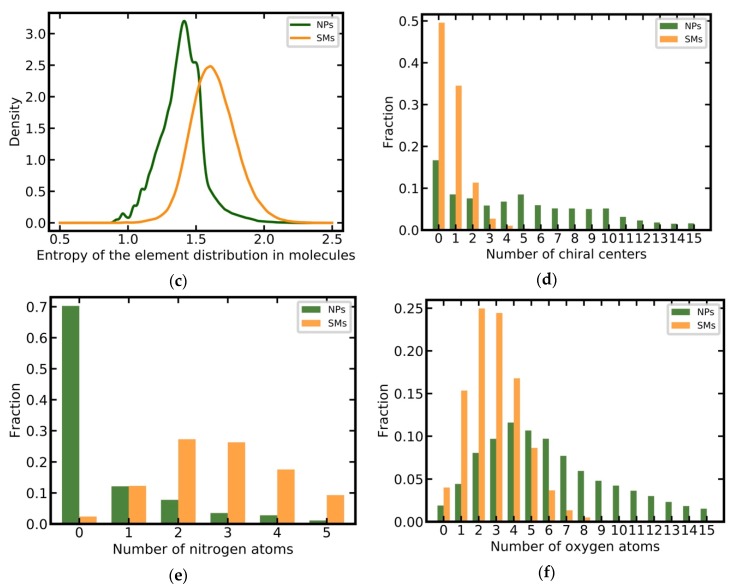
Distributions of key physicochemical properties among NPs and SMs: (**a**) Molecular weight; (**b**) log *P* (o/w); (**c**) entropy of the element distribution in molecules; (**d**) number of chiral centers; (**e**) number of nitrogen atoms; (**f**) number of oxygen atoms.

NPs have on average a higher MW than SMs (506 Da vs 384 Da) and a larger proportion of heavy compounds (38% vs. 10% of all molecules have a MW greater than 500 Da; Figure 2a). SMs have a narrower distribution of calculated log *P* values as compared to NPs (Figure 2b) but their averages are comparable (3.31 versus 3.25). SMs and NPs show clear differences in the entropy of element distributions in molecules, with NPs having, on average, a lower entropy than SMs (1.39 versus 1.63; Figure 2c). NPs tend to have more chiral centers (mean 6.66 vs. 0.75; Figure 2d), substantially fewer nitrogen atoms than SMs (mean 0.76 vs. 2.94; Figure 2e), and more oxygen atoms (mean 7.39 vs. 2.88; Figure 2f) [7,10,12,13,14,15,17].

### 3.3. Model Development and Selection

Random forest classifiers [98] were trained on three different descriptor sets: 206 two-dimensional physicochemical property descriptors calculated with MOE [47], Morgan2 fingerprints (1024 bits) [48,49] calculated with RDKit [37], and MACCS keys (166 bits), also calculated with RDKit. Model performance was characterized utilizing the Matthews correlation coefficient (MCC) [99] and area under the receiver operating characteristic curve (AUC). The MCC is one of the most robust measures for evaluating the performance of binary classifiers, as it considers the proportion of all classes in the confusion matrix (i.e., true positives, false positives, true negatives, and false negatives). The AUC was used to measure how well the models are able to rank NPs early in a list.

As reported in Table 2, the models derived from any of the three descriptor sets performed very well. The AUC values, that were obtained during 10-fold cross-validation, were between 0.996 and 0.997; the MCC values were 0.950 or higher. No noticeable increase in performance was obtained by the further increase in the number of estimators (n_estimators) and the optimization of the maximum fraction of features considered per split (max_features; data not shown). Therefore, we chose to use 100 estimators, and the square root of the number of features, as the most suitable setup for model generation.

### 3.4. Model Validation

In a first step, the performance of the selected models was tested on an independent test set. The AUC and MCC values, that were obtained for the selected models on this independent test set, are comparable with those obtained for the 10-fold cross-validation: AUC values were 0.997 for models based on any of the three types of descriptors and MCC values were 0.954 or higher.

**Figure 3:**
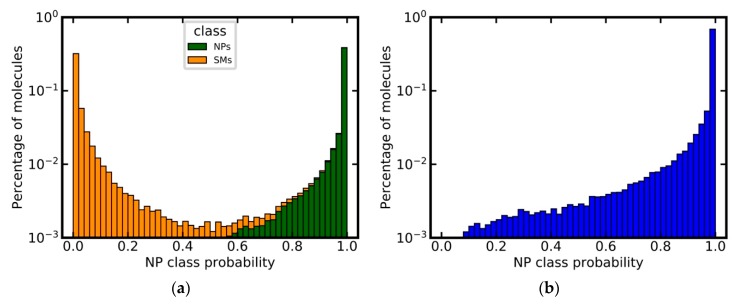
Predicted NP class probabilities distributions for (**a**) the independent test set (stacked histogram), (**b**) the DNP (after the removal of any compounds present in the training set). Note that the y-axis is in logarithmic scale.

Given the fact that the type of descriptor, used for model generation, did not have a substantial impact on model performance, we opted to select the model based on MACCS keys as the primary model for further experiments, because of its low complexity and good interpretability. This model achieved a very good separation of NPs and SMs for the independent test set, as shown in Figure 3a. Approximately 63% of all NPs were assigned an NP class probability of 1.0, whereas 51% SMs were assigned an NP class probability of 0.0. Only approximately 1% of all compounds were assigned values close to the decision threshold of 0.5 (i.e., between 0.4 and 0.6).

The model’s ability to identify NPs was also tested using the DNP as an external validation set. By definition, the DNP should consist exclusively of NPs. After the removal of any molecules present in the training data (based on canonicalized SMILES), the preprocessed DNP consisted of 60,502 compounds. Approximately 95% of these compounds were predicted as NPs by the model, demonstrating the model’s capacity to identify NPs with high sensitivity (Figure 3b).

### 3.5. Comparison of Model Performance with the NP-Likeness Calculator

We compared the performance of the model derived from MACCS keys to the NP-likeness calculator (based on the Natural-Product-Likeness Scoring System; see Introduction), which we trained and tested on the identical data sets used for the development of our models. On the independent test set, the NP-likeness calculator performed equally well as our model, with an AUC of 0.997 and an MCC of 0.959 (Table 2). Approximately 95% of all compounds of the DNP were classified as NPs (i.e., having assigned an NP-likeness score greater than 0; see Figure S1), which is comparable to the classification obtained with our model based on MACCS keys.

### 3.6. Analysis of Class Probability Distributions for Different Data Sets

**Figure 4:**
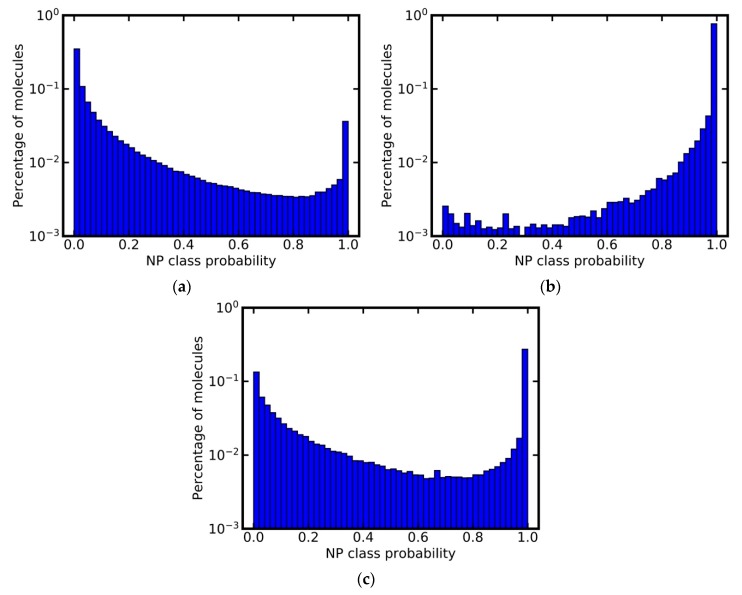
Predicted NP class probability distributions for (**a**) the ChEMBL database, (**b**) a subset of the ChEMBL database composed of molecules originating from the Journal of Natural Products, and (**c**) the natural products subset of ZINC. Note that the y-axis is in logarithmic scale.

In addition to the above experiments, we used the model based on MACCS keys for profiling the ChEMBL database and a subset thereof. The ChEMBL database [44] primarily contains SMs, and 87% of all compounds stored in ChEMBL were predicted as such (Figure 4a). Interestingly, 42,949 molecules (~3%) were assigned an NP class probability of 1.0, and therefore likely are NPs. This finding is in agreement with our previous study, which identified approximately 40,000 NPs in the ChEMBL database, by overlapping the database with a comprehensive set of known NPs [19].

A subset of the ChEMBL database containing molecules originating from the Journal of Natural Products [45] has been used as a source of genuine NPs to train models for the prediction of NP-likeness [31]. Our model based on MACCS keys predicts a small percentage of the molecules (less than 4%) in this data set as not NP-like (Figure 4b). Closer inspection of the compounds predicted as not NP-like reveals that these are, for example, SMs used as positive controls in biochemical assays. They include the drugs celecoxib, glibenclamide and linezolid, all of which are predicted with an NP class probability of 0.0. This experiment demonstrates that the classifiers can be used as powerful tools for the identification of NPs or SMs in mixed data sets with high accuracy.

A second example of a data set that by its name is assumed to consist exclusively of NPs is the natural products subset of ZINC [46]. The class probability distribution calculated for this subset however is similar to that obtained for the complete ChEMBL, indicating the presence of a substantial number of SMs (including NP derivatives and NP analogs) in this subset (Figure 4c): Only approximately 43% of all compounds in the NPs subset of ZINC were classified as NPs; around 23% were assigned an NP class probability of 1.0.

### 3.7. Analysis of Discriminative Features of Natural Products and Synthetic Molecules

The most discriminative features were determined, based on the feature_importances_ attributes computed with scikit-learn (see Methods for details). For the classifier based on MOE two-dimensional molecular descriptors, the three most important features were the number of nitrogen atoms (a large fraction of NPs has no nitrogen atom; see Figure 2e), the entropy of the element distribution in molecules (NPs have on average lower element distribution entropy than SMs; see Figure 2c), and the number of unconstrained chiral centers (NPs have on average more chiral centers than SMs; see Figure 2d). An overview of the ten most important features is provided in Table 3.

**Figure 5:**
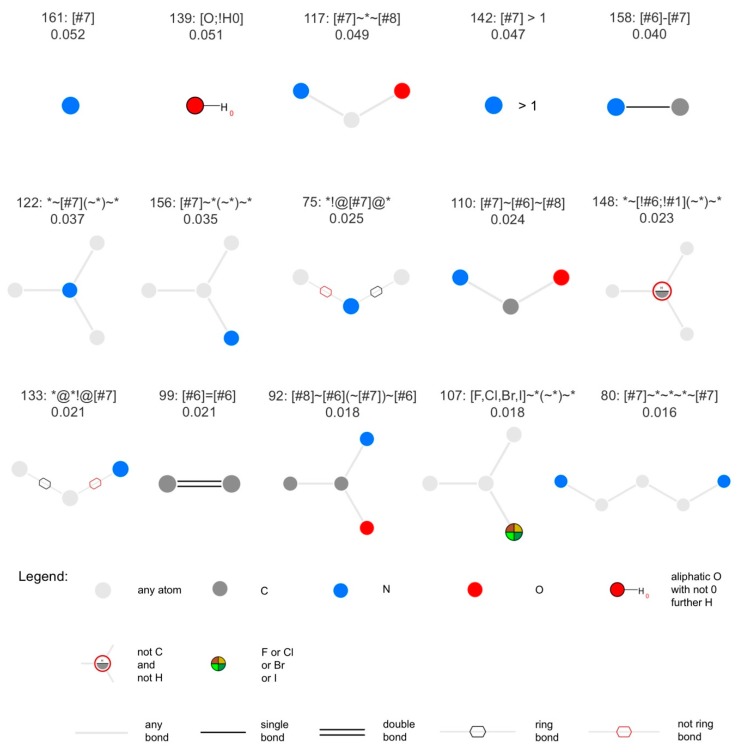
The 15 most relevant MACCS keys, sorted by decreasing feature importance. Above each diagram, the first line reports the index of the respective MACCS key and its SMARTS pattern. The second line reports the feature importance (feature_importances_ attribute). The figure was produced with SMARTSviewer [100,101].

For the classifier based on MACCS keys, the 15 most important features are reported in Figure 5. In agreement with the differences observed in the physiochemical property distributions of NPs versus SMs (see Analysis of the Physicochemical Properties of Natural Products and Synthetic Molecules), the most important MACCS keys describe the presence or absence of nitrogen atoms, such as key 161, matching molecules containing at least one nitrogen atom, key 142, matching molecules with at least two nitrogen atoms, and keys 117, 158, 122, 156, 75, 110, 133, 92 and 80, matching molecules containing specific nitrogen-containing substructures. Also several oxy gen-containing substructures are among the most important features, such as keys 139, 117, 110, 92.

### 3.8. Similarity Maps

Similarity maps [36] allow the visualization of the atomic contribution of molecular fingerprints and can be extended to visualize the “atomic weights” of the predicted probability of the machine learning model. During several test runs with different Morgan fingerprint, radii, and bit vector lengths, we identified a radius of 2 and a bit vector length of 1024 bits as the most suitable setup for generating fine-grained similarity maps. The examples of similarity maps, generated with this descriptor, and the random forest approach, are reported in Table 4 for representative molecules, none of which have been part of model training. In this similarity maps, green highlights mark atoms contributing to the classification of a molecule as NP, whereas orange highlights mark atoms contributing to the classification of a molecule as SM. As expected, the similarity maps for the NP arglabin are mostly green, whereas for the synthetic drugs, bilastine and perampanel, are mostly orange. For NP derivatives and mimetics, the similarity maps are more heterogeneous and show green, as well as orange areas. The thrombin receptor antagonist vorapaxar is a derivative of the piperidine alkaloid himbacine. Vorapaxar shares a decahydronaphtho[2,3-c]furan-1(3H)-one scaffold with himbacine, but has the piperidine ring replaced by a pyridine, besides other modifications. The similarity map generated for vorapaxar shows that the model correctly identifies the decahydronaphtho[2,3-c]furan-1(3H)-one as NP-like, whereas it associates the modified areas with synthetic molecules. In the case of empagliflozin, which mimics the flavonoid phlorozin, the model correctly recognizes the C-glycosyl moiety as NP-like, whereas other atoms in the molecule are associated with synthetic molecules.

### 3.9. NP-Scout Web Service

A web service named “NP-Scout” is accessible free of charge via http://npscout.zbh.uni-hamburg.de/npscout. It features the random forest model, based on MACCS keys for the computation of NP class probabilities and the random forest model, based on Morgan2 fingerprints (with 1024 bits) for the generation of similarity maps.

**Figure 6:**
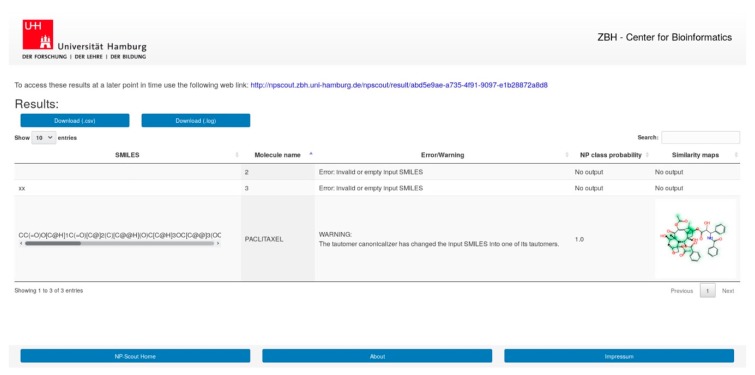
Screenshot of the result page of NP-Scout.

Users can submit molecular structures for calculation, by entering SMILES, uploading a file with SMILES or a list of SMILES, or drawing the molecule with the JavaScript Molecule Editor (JSME) [102]. The results page (Figure 6) presents the calculated NP class probabilities and similarity maps of submitted molecules in a tabular format. The results can be downloaded in CSV file format. Calculations of the NP class probabilities and the similarity maps take few seconds per compound and approximately 15 min for 1000 compounds. Users may utilize a unique link provided upon job submission to return to the website after all calculations have been completed.

## 4. Conclusions

In this work, we introduced a pragmatic machine learning approach for the discrimination of NPs and SMs and for the quantification of NP-likeness. As shown by validation experiments using independent and external testing data, the models reach a very high level of accuracy. An interesting and relevant new aspect of this work is the utilization of similarity maps to visualize atoms in molecules making decisive contributions to the assignment of compounds to either class. A free web service for the classification of small molecules and the visualization of similarity maps is available at http://npscout.zbh.uni-hamburg.de/npscout.

## Figures and Tables

**Table 1:** Size of the individual data sets prior to and after data preprocessing.

Name ^1^	Number of Molecules in SMILES Notation Successfully Parsed with RDKit	Number of Unique Molecules After Data Preprocessing	Scientific Literature and/or Online Presence
UNPD	229,140	161,228	[54,55]
TCM Database@Taiwan	56,325	45,422	[56,57]
NP Atlas	20,018	18,358	[53]
TCMID	13,188	10,918	[58,59]
TIPdb	8838	7620	[60,61,62]
Ambinter and Greenpharma NPs	7905	6680	[63,64]
AnalytiCon Discovery MEGx	4315	4063	[65]
NANPDB	6841	3734	[66,67]
StreptomeDB	3990	3353	[68,69]
NPs of PubChem Substance Database	3533	2638	[70,71]
NuBBE	1856	1637	[72,73]
Pi Chemicals NPs	1783	1511	[74]
NPCARE	1613	1479	[75,76]
NPACT	1516	1376	[77,78]
InterBioScreen NPs	1359	1116	[79]
AfroDb	954	865	[80,81]
TargetMol Natural Compound Library	850	745	[82]
HIM	1284	641	[83,84]
SANCDB	623	588	[85,86]
UEFS Natural Products	493	469	via ZINC [40,87]
p-ANAPL	538	456	[88]
NCI/NIH DTP NP set IV	419	394	[89]
HIT	707	362	[90,91]
AfroCancer	388	352	[92,93]
AfroMalariaDB	265	250	[94,95]
AK Scientific NPs	242	177	[96]
Selleck Chemicals NPs	173	163	[97]
**NP data set TOTAL**	**-**	**201761**	

^1^ UNPD: the Universal Natural Products Database; TCM Database@Taiwan: the Traditional Chinese Medicine Database@Taiwan; NP Atlas: the Natural Products Atlas; TCMID: the Traditional Chinese Medicine Integrated Database; TIPdb: the Taiwan Indigenous Plant Database; NANPDB: the Northern African Natural Products Database; StreptomeDB: Streptome Database; NuBBE: Nuclei of Bioassays, Ecophysiology and Biosynthesis of Natural Products Database; NPCARE: Database of Natural Products for Cancer Gene Regulation; NPACT: the Naturally Occurring Plant-based Anti-Cancer Compound-Activity-Target Database; AfroDb: NPs from African medicinal plants; HIM: the Herbal Ingredients in-vivo Metabolism Database; UEFS Natural Products: the natural products database of the State University of Feira De Santana; p-ANAPL: the Pan-African Natural Products Library; NCI/NIH DTP NP set IV: the NP (plated) set IV of the Developmental Therapeutic Program of the National Cancer Institute/National Institutes of Health; HIT, the Herbal Ingredients’ Targets Database; AfroCancer, the African Anticancer Natural Products Library; AfroMalariaDB, the African Antimalarial Natural Products Library.

**Table 2:** Performance of models derived from different descriptors or fingerprints.

Test Method	Metric ^1^	MOE Two-Dimensional Descriptors	Morgan2 Fingerprints (1024 Bits)	MACCS Keys	NP-Likeness Calculator
10-fold cross-validation	AUC	0.997	0.997	0.996	/
	MCC	0.953	0.958	0.950	/
Independent test set	AUC	0.997	0.997	0.997	0.997
	MCC	0.954	0.960	0.960	0.959

^1^ AUC: area under the receiver operating characteristic curve: MCC: Matthews correlation coefficient.

**Table 3:** Feature importance for the random forest classifier based on MOE two-dimensional descriptors.

Identifier Used by MOE	Feature Importance ^1^	Description
a_nN	0.103	Number of nitrogen atoms.
a_ICM	0.051	Entropy of the element distribution in the molecule.
chiral_u	0.045	Number of unconstrained chiral centers.
GCUT_SLOGP_0	0.045	Descriptor derived from graph distance adjacency matrices utilizing atomic contribution to log *P*.
SlogP_VSA0	0.044	Surface area descriptor taking into account the contributions of individual atoms to log *P*.
chiral	0.042	Number of chiral centers.
GCUT_SLOGP_3	0.036	Descriptor derived from graph distance adjacency matrices utilizing atomic contribution to log *P*.
a_nO	0.025	The number of oxygen atoms.
GCUT_PEOE_0	0.025	Descriptor derived from graph distance adjacency matrices utilizing partial equalization of orbital electronegativities charges.
SlogP_VSA1	0.024	Surface area descriptor taking into account the contributions of individual atoms to log *P*.

^1^ From the feature_importances_ attribute of the classifier based on MOE two-dimensional descriptors. The higher, the more important the feature is.

**Table 4:** Examples of similarity maps generated by the NP classifier based on Morgan2 fingerprints.

Similarity Map ^1^	Name	Source ^2^	NP Class Probability	Disease Indication	Year Introduced
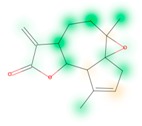	arglabin	N	1.0	anticancer	1999
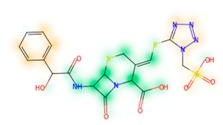	cefonicid sodium	ND	0.34	antibacterial	1984
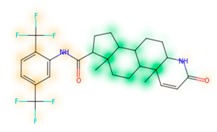	dutaseride	ND	0.18	benign prostatic hypertrophy	2001
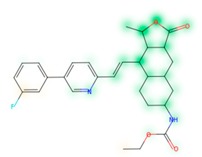	vorapaxar	ND	0.30	coronary artery disease	2014
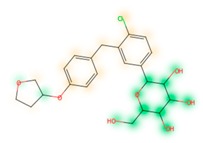	empagliflozin	S*/NM	0.67	antidiabetic (diabetes 2)	2014
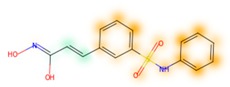	belinostat	S*/NM	0.09	anticancer	2014
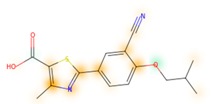	febuxostat	S/NM	0.19	hyperuricemia	2009
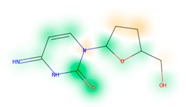	zalcitabine	S*	0.46	antiviral	1992
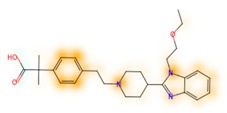	bilastine	S	0.17	antihistamine	2011
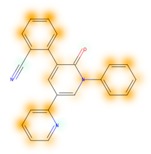	perampanel	S	0.16	antiepileptic	2012

^1^ Green highlights mark atoms contributing to the classification of a molecule as NP, whereas orange highlights mark atoms contributing to the classification of a molecule as SM. ^2^ N: Unaltered NP; ND: NP derivative; S*: Synthetic drug (NP pharmacophore); S: Synthetic drug; NM: Mimic of NP. Definitions according to ref [5].

## Supplementary Materials

The following are available online at http://www.mdpi.com/2218-273X/9/2/43/s1, Figure S1: Distribution of calculated NP-likeness scores for the DNP (after removal of any compounds present in the training set).
