# Supplementary material for: NP-Scout: Machine Learning Approach for the Quantification and Visualization of the Natural Product-Likeness of Small Molecules
Source: Biomolecules. 2019 Jan 24;9(2):43. doi: 10.3390/biom9020043 (PMC6406893; doi:10.3390/biom9020043)
Supplement: Supplementary file 1 [file biomolecules-09-00043-s001.pdf]

biomolecules

Supporting Information for

NP-Scout: Machine Learning Approach for the Quantification and Visualization of the Natural Product-Likeness of Small Molecules

Ya Chen [1], Conrad Stork [1], Steffen Hirte [1] and Johannes Kirchmair [1,2,3,*]

[1] Center for Bioinformatics (ZBH), Department of Informatics, Faculty of Mathematics, Informatics and Natural Sciences, Universität Hamburg, 20146 Hamburg, Germany; chen@zbh.uni-hamburg.de (Y.C.); stork@zbh.uni-hamburg.de (C.S.); steffen.hirte@studium.uni-hamburg.de (S.H.)
[2] Department of Chemistry, University of Bergen, 5007 Bergen, Norway
[3] Computational Biology Unit (CBU), Department of Informatics, University of Bergen, 5008 Bergen, Norway
* Correspondence: johannes.kirchmair@uib.no or kirchmair@zbh.uni-hamburg.de; Tel.: +47-5558-3464

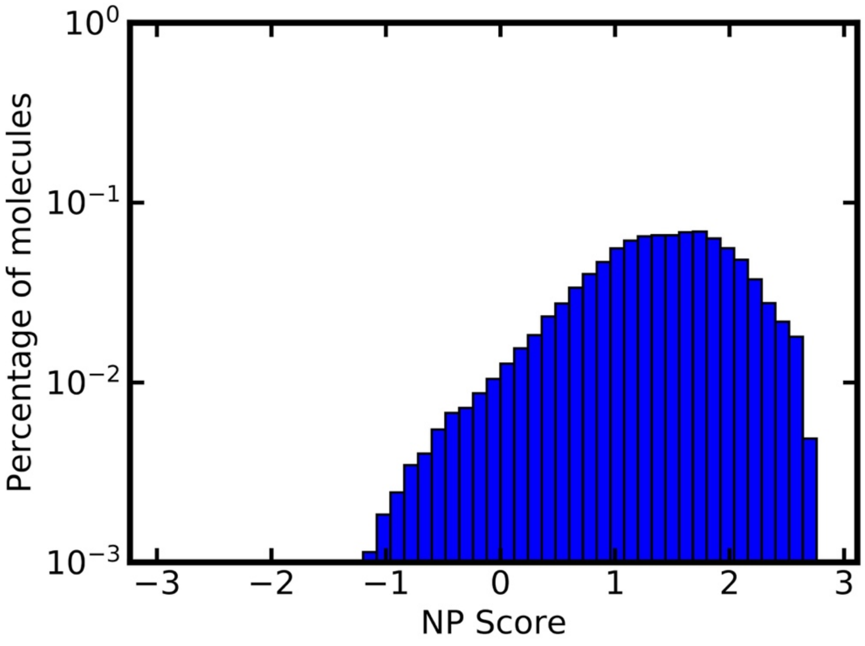

Figure S1. Distribution of calculated NP-likeness scores for the DNP (after removal of any compounds present in the training set). Note that the y-axis is in logarithmic scale.
